# PVPred-SCM: Improved Prediction and Analysis of Phage Virion Proteins Using a Scoring Card Method

**DOI:** 10.3390/cells9020353

**Published:** 2020-02-03

**Authors:** Phasit Charoenkwan, Sakawrat Kanthawong, Nalini Schaduangrat, Janchai Yana, Watshara Shoombuatong

**Affiliations:** 1 Modern Management and Information Technology, College of Arts, Media and Technology, Chiang Mai University, Chiang Mai 50200, Thailand; phasit.c@cmu.ac.th; 2 Department of Microbiology, Faculty of Medicine, Khon Kaen University, Khon Kaen 40002, Thailand; sakawrat@kku.ac.th; 3 Center of Data Mining and Biomedical Informatics, Faculty of Medical Technology, Mahidol University, Bangkok 10700, Thailand; nalini.sch@mahidol.edu; 4 Department of Chemistry, Faculty of Science and Technology, Chiang Mai Rajabhat University, Chiang Mai 50300, Thailand; janchai@g.cmru.ac.th

**Keywords:** phage virion protein, scoring card method, propensity score, interpretable model, physicochemical properties, machine learning

## Abstract

Although, existing methods have been successful in predicting phage (or bacteriophage) virion proteins (PVPs) using various types of protein features and complex classifiers, such as support vector machine and naïve Bayes, these two methods do not allow interpretability. However, the characterization and analysis of PVPs might be of great significance to understanding the molecular mechanisms of bacteriophage genetics and the development of antibacterial drugs. Hence, we herein proposed a novel method (PVPred-SCM) based on the scoring card method (SCM) in conjunction with dipeptide composition to identify and characterize PVPs. In PVPred-SCM, the propensity scores of 400 dipeptides were calculated using the statistical discrimination approach. Rigorous independent validation test showed that PVPred-SCM utilizing only dipeptide composition yielded an accuracy of 77.56%, indicating that PVPred-SCM performed well relative to the state-of-the-art method utilizing a number of protein features. Furthermore, the propensity scores of dipeptides were used to provide insights into the biochemical and biophysical properties of PVPs. Upon comparison, it was found that PVPred-SCM was superior to the existing methods considering its simplicity, interpretability, and implementation. Finally, in an effort to facilitate high-throughput prediction of PVPs, we provided a user-friendly web-server for identifying the likelihood of whether or not these sequences are PVPs. It is anticipated that PVPred-SCM will become a useful tool or at least a complementary existing method for predicting and analyzing PVPs.

## 1. Introduction

The existence of viruses that can infect and multiply only in bacteria, known as bacteriophages (or phages or bacterial virus), can be found in several environments such as soil, freshwater, and marine. Infectious phage particles are composed of a nucleic acid molecule in the form of either DNA or RNA that is surrounded by a protein coat (capsid) [1]. These bacterial viruses are very species-specific with regards to their host, which is a single bacterial strain or species. Bacteriophages irreversibly attach themselves to the surface of a susceptible host, insert their genetic information and persist using two possible strategies: lytic or lysogenic life cycle [2]. Due to their characteristics, lack of toxicity for human cells, harmless to non-specific microbes (e.g., normal flora), and their potential against antibiotic-resistant bacteria, bacteriophages are promising as alternatives to antibiotics [3]. Phage structural (virion) proteins (PVPs) including capsid proteins (coat proteins), tail proteins and phage particle enzymes, play important roles in the interaction between themselves and its host bacteria, which can be used to develop a new class of antimicrobial agents [4]. Previously, many experimental methods, such as mass spectrometry, sodium dodecyl sulfate polyacrylamide gel electrophoresis, and protein array [5,6,7], have been used for the identification of PVPs and non-PVPs. Although, the aforementioned methods are the gold standard and display high accuracy, they are too laborious and costly to be applied for large-scale analysis of PVPs from the sequence information. Hence, it is necessary to develop a computational model not only for discriminating PVPs from non-PVPs, but also for characterizing the biochemical and biophysical properties of PVPs.

Recently, many researchers have exploited various types of machine learning (ML) algorithms using sequence features to directly predict PVPs including Seguritan et al.’s method [8], Feng et al.’s method [9], PVPred [10], Zhang et al.’s method [11], PVP-SVM [12], PhagePred [13], Tan et al.’s method [14], Ru et al.’s method [15], and Pred-BVP-Unb [16], as summarized in Table 1. In 2012, Seguritan et al. [8] proposed the first predictor to identify viral structural proteins using an artificial neural network cooperating with a feature combination of amino acid composition (AAC) and protein isoelectric points. Shortly after, Feng et al. [9] established a benchmark dataset consisting of experimentally confirmed 99 PVPs and 208 non-PVPs, and developed a PVP predictor using the naïve Bayes (NB) method with AAC and dipeptide composition (DPC). Feng et al.’s method yielded an accuracy of 79.15% as assessed by the jackknife test. In the same research group [9], they also developed a new PVP predictor named PVPred [10] using support vector machine (SVM) with g-gap dipeptide composition (g-gap DPC). Moreover, this work constructed the first independent dataset consisting of 11 PVPs and 19 non-PVPs.

Until now, the state-of-the-art PVP predictors include PVP-SVM [12] and Pred-BVP-Unb [16] of which both provide high prediction accuracies of 79.80% and 83.06%, respectively, as evaluated by the independent validation test. PVP-SVM was developed by utilizing many types of sequence features, i.e., AAC, DPC, atomic composition, chain-transition-distribution and physicochemical properties, as input feature and SVM as the prediction engine. This study also gave a high number of PVPs and non-PVPs on the independent dataset (30 PVPs and 64 non-PVPs) by gathering protein sequences from the works of Ding et al. [10] and the Universal Protein Resource (UniProt). More recently, Arif et al. proposed a novel method called Pred-BVP-Unb [16] for discriminating PVPs from non-PVPs. Pred-BVP-Unb was developed by using the SVM model in conjunction with three types of protein features, i.e., composition and translation, split amino acid composition, and bi-profile position specific scoring matrix. This study also utilized feature selection (recursive feature elimination; RFE) and synthetic minority over-sampling (SMOTE) techniques for identifying an optimal feature set and overcoming the bias of an imbalanced dataset (i.e., 99 PVPs and 208 non-PVPs).

Although, Pred-BVP-Unb and other existing methods have their own merit and yielded encouraging performances with reasonably high prediction accuracies, the overall utility of the existing methods is limited in terms of few points as follows. Firstly, the underlying mechanisms of the investigated bioactivity for PVP-SVM [12] and Pred-BVP-Unb [16] affords limited interpretability for experimental scientists. Owing to the complex architecture of computational models and low interpretable features used in the study, it is not easy to identify and assess which features are beneficial for the biological activities of PVPs. As mentioned in a series of recent publications [17,18,19,20,21,22,23,24,25,26,27,28,29,30,31,32,33] and summarized in several comprehensive review papers [29,34,35,36], one of the main values of bioinformatics tools should be its ability to provide insight into mechanisms of action under study. Secondly, few existing methods were not assessed using an independent dataset, indicating that these methods might provide misleading results with overestimated accuracy. Finally, no web server was provided for many methods in this area including Seguritan et al.’s method [8], Feng et al.’s method [9], Zhang et al.’s method [11], PhagePred [13], Tan et al.’s method [14], Ru et al.’s method [15], and Pred-BVP-Unb [16]. Although, these methods have demonstrated good prediction performances, they are not generalized or transferable to researchers with informatics background who can develop in-house prediction models.

Motivated by the above mentioned limitations, we developed a novel PVP predictor named PVPred-SCM, which is an efficient and interpretable method for predicting and analyzing PVPs. PVPred-SCM was developed by using an efficient and interpretable model named the scoring card method (SCM) cooperating with only DPC to estimate the propensity scores of 400 dipeptides for predicting and analyzing PVPs. To the best of our knowledge, this study is the first in which the SCM method has been applied for predicting and analyzing PVPs. In PVPred-SCM, the propensities of 400 dipeptides to be PVPs using the statistical discrimination approach between PVPs and non-PVPs on the benchmark dataset were calculated. To enhance the prediction performance of PVPred-SCM, the propensity scores of all dipeptides were optimized using our customized genetic algorithm. Experimental results over rigorous 10-fold cross-validation and independent validation test demonstrated that PVPred-SCM had promising performance, compared to existing SVM-based methods with various types of protein features. To identify unknown sequences, PVPred-SCM based on the weighted-sum function can easily be adopted using a single threshold for discriminating PVPs from non-PVPs. Furthermore, the propensity scores of amino acids were investigated to elucidate the biochemical and biophysical properties of PVPs by using the correlation coefficient (R) between the propensity scores and the physicochemical properties (PCPs) from AAindex [37]. Finally, PVPred-SCM was developed as a user-friendly and publicly accessible web server that allows robust predictions to be made without the need to develop in-house prediction models.

## 2. Results

**Figure 1:**
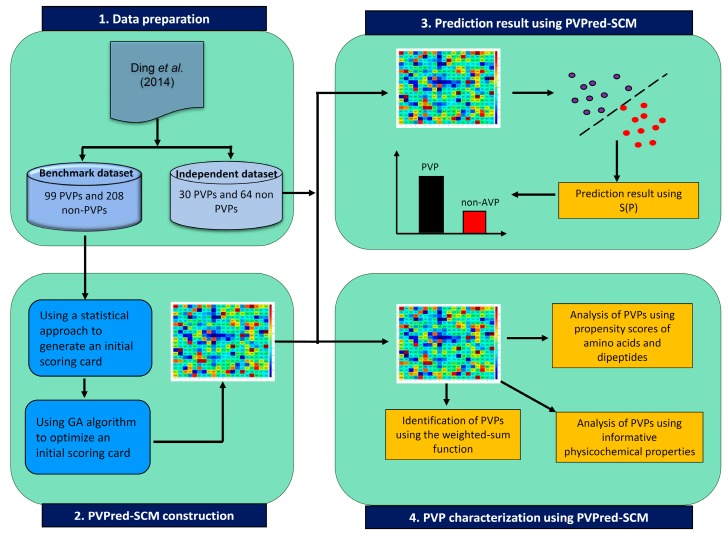
Schematic framework of PVPred-SCM for prediction and analysis of phage virion proteins (PVPs).

In this study, PVPs and non-PVPs were predicted by the proposed method PVPred-SCM. Figure 1 summarizes the workflow of the computational approach of PVPred-SCM. It can be seen that four steps are involved in the development of this method as follows: (i) collecting both benchmark and independent datasets, (ii) calculating init-DPS using a statistical approach, (iii) optimizing init-DPS to obtain opti-DPS using the genetic algorithm (GA), (iv) PVP prediction, and v) PVP characterization using the propensity scores of dipeptides. Finally, to serve easy and rapid classification of query protein sequence, PVPred-SCM was utilized as a free prediction web server for discriminating PVPs and non-PVPs.

### 2.1. Prediction Performance

In this study, we performed both 10-fold cross-validation and independent validation test on the benchmark and independent datasets to demonstrate the efficient and effective prediction of the proposed method. To make a fair comparison with the existing PVP predictors [9,10,12,13,14], the same benchmark and independent datasets that have been used in previous studies [12] were used to develop our proposed model. Due to the non-deterministic characteristic of the GA algorithm [26,32], ten SCM models in conjunction with ten different optimized dipeptide propensity scores (opti-DPS) [21,22,23,24,25,26,27,38] were performed to generate ten different prediction results. Table 2 and Table 3 list the performance comparisons of ten independent runs evaluated by 10-fold CV and independent validation test, respectively.

**Figure 2:**
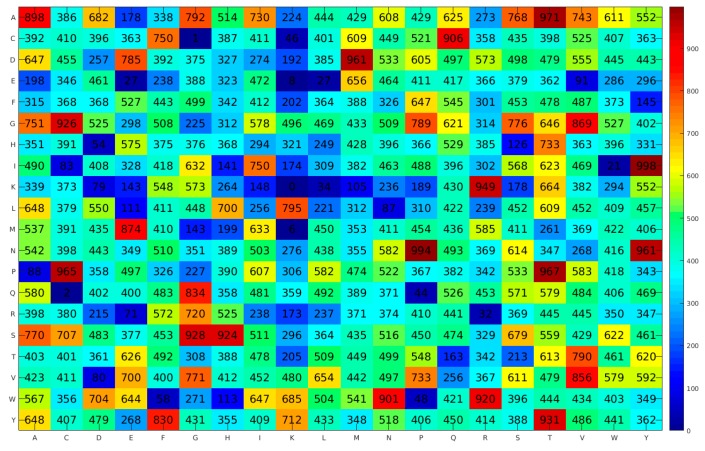
Heatmap of dipeptide propensity scores obtained from the PVPred-SCM method.

As seen in Table 2, the average prediction results of the ten individual experiments over 10-fold CV were 93.18% accuracy (ACC), 95.74% sensitivity (SN), 91.95% specificity (SP), 0.858 Matthews coefficient correlation (MCC), and 0.954 area under the receiver operating characteristic (ROC) curve (auROC). It could be noticed that the three-top ranked experiments exhibiting the highest prediction results (Ac, MCC, and auROC) were experiments #3 (95.11%, 0.894, and 0.966), #5 (94.44%, 0.882, and 0.960), and #10 (93.82%, 0.866, and 0.960), respectively. Meanwhile, Table 3 shows that the average prediction results of the ten individual experiments over the independent validation test were 74.47% ACC, 71.67% SN, 75.78% SP, 0.455 MCC, and 0.768 auROC. The three-top ranked experiments having highest prediction results (ACC, MCC, and auROC) were experiments #9 (77.66%, 0.523, and 0.781), #3 (76.60%, 0.482, and 0.781) and #5 (76.60%, 0.461, and 0.793), respectively. Although, experiment #9 was not in the three-top ranked experiments over 10-fold CV, it provides a promising result in terms of ACC, MCC, and auROC with 92.52%, 0.846, and 0.948, respectively, which was not significantly different from the result of experiment #3 (95.11%, 0.894, and 0.966). Moreover, due to the fact that the independent test was the most rigorous cross-validation method to demonstrate the robustness and reliability of the model in real-world applications [17,18,19,20,28,29,31,33,39,40,41], it could be noted that experiment #9 provided an important contribution to PVP prediction. For convenience, the best PVP predictor based on the SCM method in conjunction with the propensity scores of dipeptides from experiment #9 would be referred to as PVPred-SCM. Meanwhile, the opti-DPS from experiment #9 was also used for providing a good understanding of the biochemical and biophysical properties of PVPs, as depicted in Figure 2.

### 2.2. Contribution and Effectiveness of Dipeptide Propensity Scores

As mentioned in Table 2 and Table 3, the opti-DPS obtained from experiment #9 outperformed the others in terms of Ac and MCC over the independent validation test. In this section, to investigate the effectiveness of the opti-DPS, we conducted a performance comparison between PVPred-SCM based on opti-DPS and initial dipeptide propensity (init-DPS) as assessed by the 10-fold CV and independent validation test, shown in Table 4. The init-DPS was calculated using the normalized dipeptide composition, while the opti-DPS was generated by optimizing the init-DPS with the GA algorithm as described in the scoring card method section.

**Figure 3:**
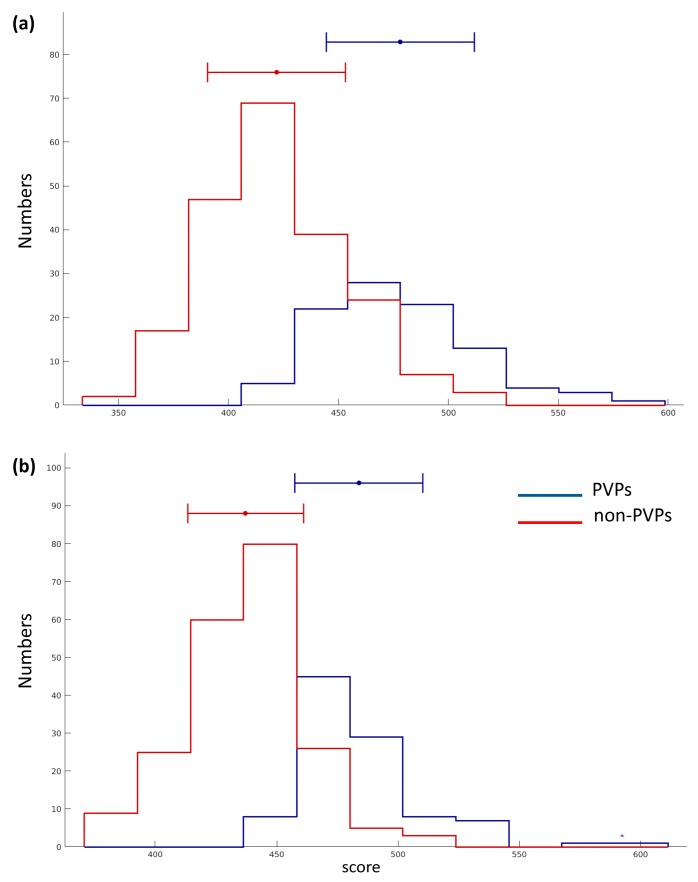
The histogram of scores of PVPs and non-PVPs derived from PVPred-SCM on the benchmark dataset by using initial (**a**) and optimized (**b**) dipeptide propensity scores, respectively.

From Table 4, PVPred-SCM based on the opti-DPS could enhance the values of ACC, SN, and MCC by 2%, 23%, and 11%, respectively, suggesting that opti-DPS provided more discriminative power than init-DPS. Moreover, the histogram was also used to demonstrate the discriminative power of opti-DPS and init-DPS for PVP prediction as depicted in Figure 3a,b, respectively, where blue and red lines, represent the score distribution of PVPs and non- PVPs, respectively. In practice, more overlap between the red and blue bars indicates that the feature was less capable in PVP prediction. Thus, it could be clearly seen in Figure 3 that opti-DPS was efficient and effective as the input feature for discriminating PVPs from non-PVPs.

### 2.3. Comparison with Existing Methods

As mentioned in the Introduction section, many research groups have made efforts to develop computation models for PVP prediction. To demonstrate the efficiency and strength of the proposed model, we made a comparison between PVPred-SCM and previously developed PVP predictors on the same benchmark (99 PVPs and 208 non-PVPs) and independent (30 PVPs and 64 non-PVPs) datasets, i.e., Feng et al.’s method [9], PVPred [10], PVP-SVM [12], PhagePred [13], and Tan et al.’s method [14], by performing both 10-fold CV and independent validation tests. As noticed from Table 5, there were two different experimental designs: (i) the prediction models were assessed using only 10-fold CV, i.e., Feng et al.’s method and PhagePred, and (ii) the prediction models were assessed on both 10-fold CV and independent tests, i.e., PVPred, PVP-SVM, and Tan et al.’s method.

Table 5 lists the performance comparisons between PVPred-SCM and the existing methods over the 10-fold CV and independent validation test. From Table 5, the highest prediction result over 10-fold CV (Ac, MCC) of (98.05%, 0.963) was achieved by PhagePred, while the second and third highest prediction results of (92.5%, 0.846) and (87.95%, 0.761) were achieved by PVPred-SCM and Tan et al.’s method, respectively. Based on the performance comparisons over an independent validation test, PVP-SVM outperforms other PVP predictors, while PVPred-SCM achieved very comparable or slightly worse than PVP-SVM in terms of Ac (79.80% vs. 77.66%) and MCC (0.531 vs. 0.523). On the other hand, the SN value obtained from PVPred-SCM was approximately 10.0% higher than that of PVP-SVM.

To validate the robustness of the proposed PVPred-SCM, we further tested and compared its performance using another independent dataset derived from Zhang et al.’s method [11] (containing 68 PVPs and 92 non-PVPs). Table S1 lists the results of this independent test. As noticed in Table 1, PVPred-SCM outperformed the two existing methods, i.e., Feng et al.’s method and PVPred. Meanwhile, Zhang et al.’s method obtained better prediction results than PVPred-SCM because the classifier employed was developed using a stack-based ensemble method in conjunction with various sequence features [11].

With regard to the performance comparison between PVPred-SCM and the existing methods as discussed above, apparently, the best performance of PVPred-SCM contributed to the best SN and the runner-up ACC and MCC. This result demonstrates that the proposed PVPred-SCM could be reliable for identifying truer PVPs (true positives) and fewer false positives. These prediction results revealed that the novelty and significance of PVPred-SCM was as follows: (i) amongst various types of ML algorithms and features employed in developing PVP predictors, the SCM method in conjunction with the propensity scores of dipeptides has never before been employed in developing a PVP predictor. Previously, many studies reported that this feature has been successfully exploited to predict and analyze many protein functions [21,22,23,24,25,26,27,32]; (ii) PVPred-SCM was constructed using only a single type of protein feature and the prediction results of unknown proteins were obtained by using the easy-to-use weighted-sum function as illustrated in the Scoring Card Method Section. It could be indicated that PVPred-SCM can be easily understood and manipulated by experimental scientists who want to use the prediction method without the interest to follow the mathematical and statistical details. (iii) Most of the existing PVP predictors [9,10,12,13,14] developed using NB and SVM methods were used for increasing prediction results. However, the underlying biochemical and biophysical implications remained hard to interpret. Therefore, the interpretability of the proposed method with a satisfied prediction result is a more useful and practical approach. We thus claimed that PVPred-SCM was very promising, as compared to PVP-SVM (77.66% vs. 79.80%) considering its simplicity, interpretability, and implementation.

### 2.4. Identification of Phage Virion Proteins

**Figure 4:**
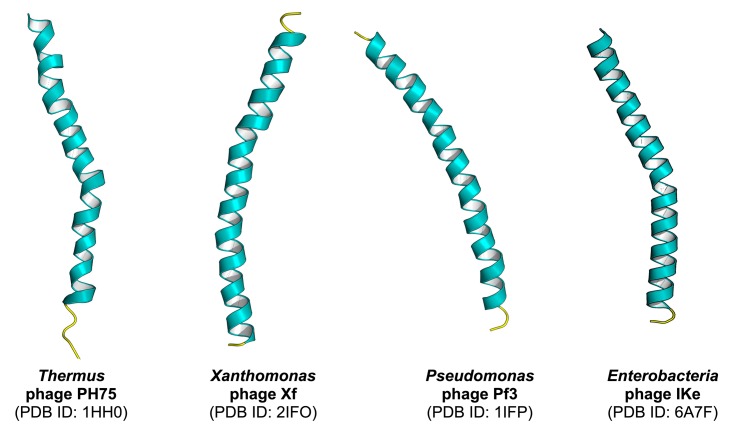
Structures of selected PVPs elucidated via the fiber diffraction method. Each structure is labeled by a common name followed by the Protein Data Bank identification number (PDB ID) in parenthesis on the subsequent line.

One of the most important benefits of PVPred-SCM is to facilitate researchers by easily identifying their desired protein with very simple weighted-sum function (S(P)) [21,25]. In this study, PVPred-SCM was used to calculate the PVP scores on the benchmark dataset for finding a new PVP, which has not yet been experimentally characterized, where the PVP scores are calculated using the scoring function S(P) as illustrated in the Scoring Card Method Section. Table 6 lists the top ten potential proteins to be PVPs having the highest PVP scores along with their name, PDB ID, UniProt ID, and source. The mean, minimum, and maximum PVP scores on the benchmark dataset were 452.27, 370.75, and 605.69, respectively. As noticed in Table 6, all top ten proteins had PVP scores with greater than 461.75, where P was classified as a PVP when the PVP score was greater than 461.75; otherwise, P was classified as non-PVP. It could be noted that the selected ten proteins had high potential to be PVPs. Among the top ten proteins having the highest PVP scores, there were only four PVPs that had their structures elucidated via the fiber diffraction method, i.e., Capsid protein G8P, Capsid protein G8P, G VIII capsid protein Precursor, and Major coat protein as shown in Figure 4, while the structures of the rest six PVPs have not yet been determined. As noticed in Table 6, most of the top ten potential phage virion proteins with the highest PVP scores played an important role in the same function such as capsid or coat proteins. Remarkably, among the mentioned top ten potential phage virion proteins, there were two PVPs appearing to be the major coat protein (gene VIII product; G8P or pVIII; p8) subunit of filamentous phage PH75 and phage Xf. The p8 subunit is a helix consisting of around 50 amino acid residues, which form a filament-like capsid that is involved with phage virion assembly [42]. Therefore, for further analysis, capsid proteins were chosen in the characterization of PVPs. In practice, bacteriophage capsids are present in a variety of shapes, ranging from icosahedral to highly elongated [43].

### 2.5. Analysis of Phage Virion Proteins Using Propensity Scores of Amino Acids and Dipeptides

The characterization and analysis of feature importance can provide a unique understanding of PVPs. In this study, the propensity score of amino acids and dipeptides were used to identify the important biochemical and biophysical properties of PVPs. Table 7 and Figure 2 list the propensity scores of amino acids and dipeptides, respectively, derived from the benchmark dataset consisting of 99 PVPs and 208 non-PVPs. The propensity scores of dipeptides were obtained from the opti-DPS from experiment #9, while the propensity scores of amino acids (PVP score) were calculated from such opti-DPS using a straightforward statistical approach as mentioned in our previous studies [26,32]. Amino acids and dipeptides with the highest propensity scores are the most important elements in PVPs.

As seen in Table 7, Ala (529.50), Thr (511.43), Val (506.88), Gly (506.68), and Ser (504.63) were the five top-ranked amino acids present in PVPs, while Leu (395.85), Arg (383.15), His (378.45), Glu (358.93), and Lys (310.90) were the five top-ranked informative amino acids present in non-PVPs. Interestingly, the five top-ranked amino acids representing PVPs and non-PVPs were significantly different between the classes at the level of *p* < 0.05, except for Leu (0.160). These results revealed that the informative amino acids were beneficial for PVP prediction. Based on the propensity scores of dipeptides as shown in Figure 2, the twenty top-ranked dipeptides represented in PVPs included IY, NP, AT, PT, PC, DM, NY, KR, YT, SG, GC, SH, WR, CQ, WN, AA, ME, GV, VV, and QG with propensity scores of 998, 994, 971, 967, 965, 961, 961, 949, 931, 928, 926, 924, 920, 906, 901, 898, 874, 869, 856, and 834, respectively. Meanwhile, the twenty top-ranked dipeptides represented in non-PVPs included KK, CG, QC, MK, EK, IW, EE, EL, RR, KL, QP, CK, WP, HD, WF, RE, KD, VD, IC, and LN with propensity scores of 0, 1, 2, 6, 8, 21, 27, 27, 32, 34, 44, 46, 48, 54, 58, 71, 79, 80, 83, and 87, respectively.

Interestingly, our results from the propensity scores of amino acids were consistent with the computational results made by Ding et al. [10]. Their results were derived using the analysis of variance (ANOVA) and visualized via a heat map. This study reported that Ala, Gly, Pro, Ser, Thr, and Val were preferred in PVPs. Glu, Lys, Leu, and Arg were preferred in non-PVPs. Based on our analysis results, the ranks of propensity scores for Ala, Thr, Val, Gly, and Ser were 1, 2, 3, 4, and 5, while the ranks of propensity scores for Leu, Arg, Glu, and Lys, were 16, 17, 19, and 20, respectively as shown in Table 7. Furthermore, Lin et al. [44] analyzed the amino acid composition of filamentous bacterial virus xf (*Xanthomonas oryzae*) coat protein, which showed that His, Cys, and Phe were absent from the xf protein, indicating that these three amino acid were not preferred in PVPs. Apparently, the ranks of propensity scores for His, Cys, and Phe were 18, 13, and 15. 

### 2.6. Analysis of PVPs Using Informative Physicochemical Properties

In this section, the PVPred-SCM method was exploited to analyze the crucial physicochemical properties (PCPs) of PVPs. Previously, many studies have reported that the biochemical and biophysical properties such as side-chain [45], alpha-helix propensity [46,47], and hydrophobicity [48] affect the biological activities of PVPs. To analyze the important characteristics of PVPs, Pearson correlation coefficient (R) between the propensity scores of amino acids and the PCPs in the AA index were used to identify informative PCPs that were useful in the analysis of PVPs. As seen in Table 8, the three selected PCPs were QIAN880126 (R = 0.502), WOLR790101 (R = 0.484), and side-chain (R = −0.516), respectively, according to the important biochemical and biophysical properties for PVPs as mentioned above. Additional details of the top ten informative PCPs having highest and lowest R values are listed in Tables S2 and S3. The analysis results cooperating with the propensity scores of amino acids and the three selected PCPs are discussed below.

KOEP990101, which can be described as the “Alpha-helix propensity” [49], had a high positive correlation (R = 0.502). Structure-based conformational preferences of amino acids were reported based on the knowledge of the template backbone on a physical potential energy function, which is composed of a Lennard-Jones potential, electrostatics based on the Coulomb potential, and interatomic contact areas [50]. The high positive correlation suggests that PVPs favor a high alpha-helix propensity. As noticed in Table 8, there were two amino acids, i.e., Gly and Thr, found in the five top-ranked amino acids having the highest propensity scores as well as alpha-helix propensity. The ranks of propensity scorers (PS, alpha-helix) for Gly and Thr were (4, 1) and (2, 3), respectively. Although, the rank of Ala was (1, 12), Pace et al. found that the estimated differences in free energy or Δ(ΔG) values in kcal/mol of Ala has been set to zero because it is usually the amino acid with the most favorable helix propensity [46]. They also mentioned that the helix propensity was an important contribution to protein stability. In the backbone effect, a α-helix was stabilized by a strong H-bond and Van der Waals interactions. This study reported that if the α-helix was substituted with Ala, favorable interactions were observed and the helix was more stable than the coil formation. However, if the α-helix was composed of Gly, unfavorable interactions were observed, which resulted in the coil formation being more stable than the helix due to a large reduction in phi–psi space available to residues. Meanwhile, the H-atom of Gly was replaced by a CH_3_ in Ala; this is almost entirely an effect of the CH_3_ group on the conformational entropy of the backbone in the coil formation. On the other hand, the CH_3_ group of Ala experiences no loss in conformational entropy when a randomly coiled polypeptide folds to an α-helix.

It could be noticed that the property of side-chain [47] of amino acid had a high negative correlation (R = −0.516), indicating that PVPs favor small amino acids. As seen in Table 8, five top-ranked important amino acids present in PVPs were Ala, Thr, Val, Gly, and Ser, while according to the property of side-chain [50], such five amino acids (PS, side-chain) were ranked at (1,19), (2,15), (3,16), (4,20), and (5,18), respectively. Our analysis result was well consistent with the work of a previous study [45]. In this study, Kuzmicheva et al. established a fusion phage protein containing the β-galactosidase-binding peptide at the N-terminal region by random mutations in amino acids 12–19 adjoining the guest peptide. Based on their experimental results, they suggested that small amino acids played a vital role in ensuring a high binding affinity for the domain C of the phage major coat protein. For the case under their discussion, it could be found that large amino acids tended to have a deleterious effect on the affinity of the guest peptides towards its target. In this study, they mentioned two reasons that form important chemical properties of small amino acids for the phage with the highest affinity: (i) low radius of octapeptide constituting domain C could constrain alpha helix regions and (ii) low steric hindrances leading to low conformation number (i.e., smaller side chain has lower steric hindrances).

In addition, WOLR790101, which can be described as the “Hydrophobicity index” [47], had a high positive correlation (R = 0.484). The hydrophobicity index is a measure of the relative hydrophobicity of each amino acid, which correlated to their solubility in water. Amino acids with hydrophobic side chains also show positive correlation with the hydrophobicity index. As could be seen in Table 8, there were three important amino acids found in the ten top-ranked highest propensity scorers and hydrophobicity index, i.e., Ala (1, 5), Val (3, 4), and Gly (4, 1). Previously, various experimental studies have mentioned that amino acid substitution could affect the stability of procapsids and virions of bacteriophages. For example, Roth et al. constructed minimized-M13 filamentous bacteriophage major coat proteins (P8) to study the minor contributors to the phage assembly process. The result showed that mimi-P8 containing only 50-residues, which comprised of 4 Gly, 37 Ala, and 9 larger residues that were required for phage coat incorporation [51]. In bacteriophage P22, the coat protein E-loop residues Glu52, Glu59, and Glu72 were hypothesized to play a role in stabilizing the capsid by making salt bridges with an adjacent subunit, the P-domain residues R102, R109, and K118, respectively within a capsomer. To verify this hypothesis, a mutant coat protein was constructed by Ala substitution at the E-loop site to remove the charge from Glu without increasing the electrostatic repulsion. The result found that the number of virions assembled within the wild-type coat protein decreased to 50% at 2 M urea, while phages assembled with mutant coat protein showed a 50% survival rate in a higher concentration of urea. It could be noted that the substitution of Glu with Ala increased the stability of procapsids and virions of bacteriophages. Although electrostatic repulsion could cause destabilization between the E-loop residue and spine helix, hydrophobic interaction could relieve this effect by balancing the repulsive electrostatic interactions [48]. As noticed in Table 8, the ranks of propensity scores (PS, hydrophobicity index) for Glu and Ala were (18, 19) and (1, 5), respectively, while the propensity scores of charged amino acid side chains of Lys, Glu, His, Arg, and Asp were ranked at 20, 19, 18, 17, and 12, respectively.

### 2.7. Web Server Implementation

**Figure 5:**
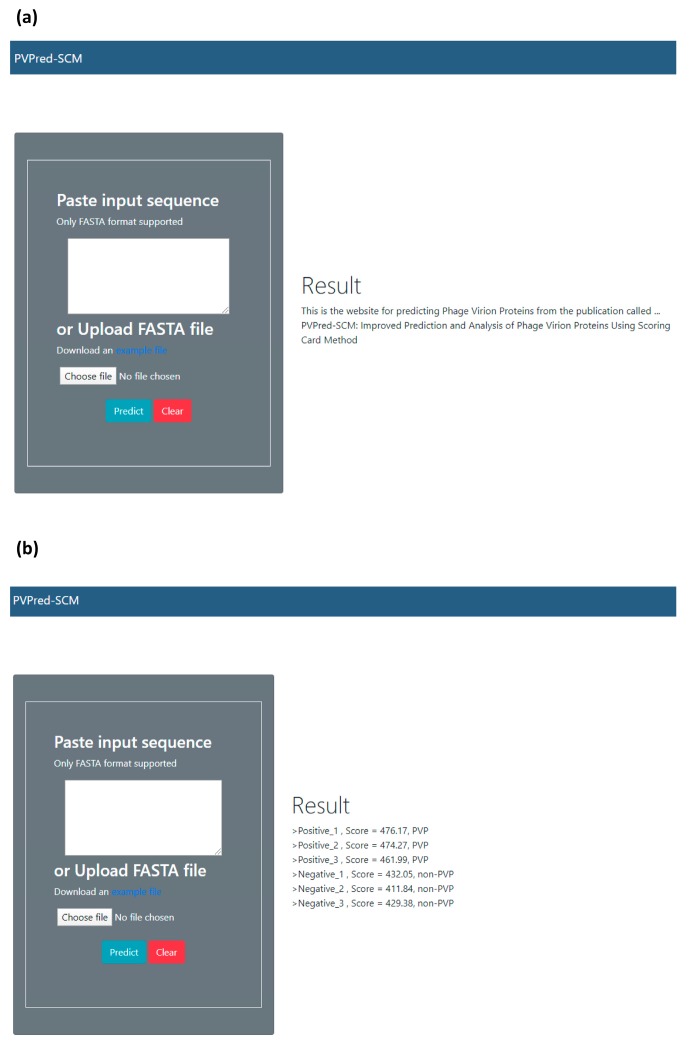
Screenshots of the PVPred-SCM web server before (**a**) and after (**b**) submission of a query protein. Prediction results are represented with the PVP scores derived from the scoring function (S(P)) and predicted classes.

In an effort to facilitate high-throughput prediction of PVPs, the best predictive model was deployed as a PVPred-SCM web server and is made freely available online at http://camt.pythonanywhere.com/PVPred-SCM where users can submit query protein sequences for determining the likelihood of these proteins being PVPs. Below, we provide step-by-step guidelines on how to use the PVPred-SCM web server in order to obtain the desired results. Firstly, the user opens the web server at http://camt.pythonanywhere.com/PVPred-SCM and the user will see the top screen of PVPpred on the user’s computer screen, as shown in Figure 5. Secondly, the user enters the query sequence into the text box or uploads a FASTA file by clicking on the “Choose file” button. Thirdly, the user clicks on the “Submit” button in order to start the prediction process. Finally, after finishing the prediction process, the results are outputted as shown on the right-hand side of the web server. The user can see examples of FASTA-formatted sequences by clicking on the “example file” button. In fact, user-friendly and publicly accessible web-servers that can display the findings manipulated by users according to their needs, might significantly enhance their impact, driving medicinal chemistry into an unprecedented revolution [52,53,54,55,56]. Keeping this point in our mind, we shall make efforts in our future work to provide a web-server with such functionality.

### 2.8. Reproducible Research

To guarantee and ensure the reproducibility of the proposed model, herein, all codes and datasets used in the construction of the predictive models are available on GitHub at https://github.com/Shoombuatong2527/PVPred-SCM.

## 3. Materials and Methods

In order to establish a robust and interpretable sequence-based tool for modeling the investigated PVPs, we followed the six prime keys as mentioned in a series of recent publications [17,18,19,20,21,22,23,24,25,26,27,28,29,30,31,32,33] and summarized in several comprehensive review papers [29,34,35,36]: (i) establishing a reliable dataset that contains experimentally validated sequences for training and validating the model; (ii) representing protein sequences with interpretable features; (iii) developing interpretable learning algorithms so as to allow the interpretation of important features responsible for the biological activity; (iv) assessing the prediction model using standard cross-validation tests; (v) constructing a user-friendly web-server for obtaining the prediction without the need to understand complex mathematical and statistical details; and (vi) analyzing and characterizing the important features derived from the developed model to provide a better understanding of the biophysical and biochemical properties of proteins. Figure 2 shows the workflow of PVPred-SCM, which works in predicting and analyzing PVPs.

### 3.1. Dataset Preparation

One of the most crucial steps is to establish a reliable and stringent benchmark dataset to train and validate the proposed method. To make a fair comparison with existing methods, the same benchmark ($S_{\mathrm{TR}}$) and independent ($S_{\mathrm{TS}}$) datasets derived from the work of [12] were taken to develop and validate the proposed model, respectively. These two datasets were specifically used to develop PVP predictors for discriminating PVPs from non-PVPs. The main reason for selecting these two datasets was that the protein sequences were non-redundant, while protein sequences with more than 40% similarity were removed from the datasets, thereby avoiding misleading results with overestimated accuracy. In this study, the benchmark dataset consists of 99 PVPs and 208 non-PVPs, while the independent dataset consists of 30 PVPs and 64 non-PVPs. We noted that none of the PVPs in the independent dataset are identical to PVPs in the benchmark or training dataset. The benchmark ($S_{\mathrm{TR}}$) and independent ($S_{\mathrm{TS}}$) datasets used in this study can be summarized by the following formula:

(1) $$S_{\mathrm{TR}}=S_{\mathrm{TR}}^{+}\cup S_{\mathrm{TR}}^{-}$$

(2) $$S_{\mathrm{TR}}=S_{\mathrm{TR}}^{+}\cup S_{\mathrm{TR}}^{-}$$

where $S^{+}$ and $S^{-}$ represent peptide sequences of PVPs and non-PVPs, respectively, while the symbol ∪ represents the union from the set theory.

### 3.2. Feature Representation

Given a protein sequence (P), it can be represented as:

(3) $$P=p_{1}p_{2}p_{3}\ldots p_{1N}$$

where $p_{i}$ and N denote the *i*^th^ residue in the protein P and the peptide length, respectively. Note that the residue $p_{i}$ is in the set of natural amino acid, i.e., A, C, D, E, F, G, H, I, K, L, M, N, P, Q, R, S, T, V, W, and Y. In the development of a sequence-based predictor for characterizing and analyzing the biophysical and biochemical property of peptides, one of the most crucial aspects is how to best represent the peptides in such a way as to afford a comprehensive and proper description of the feature that could well reflect their functions. Until now, various convenient tools have been developed for representing a protein sequence using a fixed-length feature vector, such as BioSeq-Analysis2.0 [57], iFeature [58], protr/ProtrWeb [59], etc. Many studies have reported that DPC is one of the most interpretable and effective features for predicting and analyzing various types of protein and peptide functions [12,17,18,19,20,21,22,23,24,25,26,28,29,30,31,32,33,39,40,60,61,62,63,64,65,66]. Basically, DPC are the proportion of dipeptide in a peptide sequence P that are expressed as a fixed length of 400. Thus, in terms of the DPC feature, a protein sequence P can be expressed by a vector with 400D (dimension), as formulated by:

(4) $$P={\left[{\mathrm{dp}}_{1},{\mathrm{dp}}_{2},\ldots ,\;{\mathrm{dp}}_{400}\right]}^{T}$$

where T is the transposed operator, while dp_1_, dp_2_..., dp_400_ are occurrence frequencies of the 400 native dipeptides, respectively, in a protein sequence P. 

### 3.3. Scoring Card Method

Amongst various types of machine learning algorithms that are available, SCM method has been successfully implemented to predict and characterize many protein functions [21,22,23,24,25,26,27] by using only the sequence information. SCM method is an efficient and simple approach that performs well not only for predicting proteins by using an efficient function, but also for gaining insight into the characteristic of proteins based on the propensity scores of dipeptides and amino acids. The advantages of the SCM method are threefold. Firstly, the prediction procedure of SCM method is significantly simpler than the SVM model. For the prediction of unknown proteins, the SCM method exploits a weighted summation of opti-DPS and the occurrence frequencies of the 400 native dipeptides. Secondly, the propensity scores of amino acids and dipeptides play an important role in understanding the underlying biophysical and biochemical properties governing biological activities of proteins and peptides. Finally, the SCM model is a general-purpose method for predicting protein and peptide functions by using an efficient function. Therefore, in this study, the SCM method was utilized for developing the PVP predictor named PVPred-SCM. More details for the SCM method were described in our previous studies [26,27,32]. Herein, the basic concepts and associated parameter optimizations for the SCM method is briefly described as follows:

Step 1. Preparing a benchmark dataset ($S_{\mathrm{TR}}$) for training and developing PVP prediction. 

Step 2. Generating init-DPS consisting of 400 propensity scores of dipeptides and then normalizing such propensity scores into [0,1000], where dipeptides with the highest propensity scores are the most important residues in PVPs. It could be stated that the init-DPS is derived from the normalized dipeptide composition.

Step 3. Calculating propensity scores of amino acid AA_i_ by averaging 40 propensity scores of dipeptides that contain AA_i_.

Step 4. Optimizing init-DPS by using the GA method. In this study, the fitness function of the GA method was used to maximize both the prediction performance in terms of auROC and the Pearson’s correlation coefficient (R) between init-DPS and opti-DPS. To obtain the best opti-DPS, the fitness function (Fit-Funt) is assessed by 10-fold CV defined as follows:

(5) $$\mathrm{Fit}-\mathrm{Funt}=W_{1}\times \mathrm{auROC}+W_{2}\times R$$

where the values of $W_{1}$ and $W_{2}$ are 0.9 and 0.1, respectively. The detail for the optimization of these two parameters was described in our previous studies [26,27,32].

Step 5. Predicting an unknown protein sequence (P_unknown_) by using the scoring function (S(P)). The procedure of the prediction of an unknown sequence is briefly described as follows (i) choosing the best opti-DPS with the highest score derived from Fit-Funt and (ii) calculating the summation of 400 propensity scores ($S_{i}$) and frequencies ($w_{i}$) of dipeptides, predicting P_unknown_ as PVP if S(.) is greater than the threshold value, otherwise P_unknown_ is predicted as non-PVP.

(6) $$S(P_{unknown})=\overset{400}{\underset{i=1}{\sum }}w_{i}S_{i}$$

### 3.4. Characterization of Phage Virion Proteins

In this work, we performed the propensity score of amino acids and dipeptides using the SCM method to provide a better understanding of the biophysical and biochemical properties of PVPs. The calculation of propensity scores of amino acids and dipeptides was described in the above section. The values of propensity scores represent the influence of each amino acid and dipeptide on the biological, functional, and structural properties of proteins.

Furthermore, the informative physicochemical property (PCP) of amino acids was identified using PVPred-SCM. PCPs are one of the most intuitive features associated with biophysical and biochemical reactions. Previously, our studies utilized these features for predicting and analyzing various functions of proteins and peptides from primary sequences [17,19,20,26,28,29,32,33,67]. In fact, there are 544 PCPs of amino acids extracted from the amino acid index database (AAindex) [37], which is a collection of published literature as well as different biochemical and biophysical properties of amino acids. Each PCP consists of a set of 20 numerical values for amino acids. PCPs with not applicable (NA) as their amino acid indices were excluded, and a total of 531 PCPs were further used in this study.

The procedure of determining informative PCPs using PVPred-SCM consists of two main steps as follows: (i) calculating the R value between the propensity scores of amino acids and the 531 PCPs. PCPs with highest absolute R values indicate that these PCPs are highly correlated with the biological, functional, and structural properties of PVPs, and (ii) PCPs with an absolute R value of greater than 0.5 are preferred as a candidate for PVP characterization [22,23,25].

### 3.5. Performance Evaluation

Cross-validation of the proposed method is an essential step in the development of computational models [29,35,36,68,69,70,71,72,73,74]. These cross-validation procedures were used in order to assess the success and error rates of the proposed method. In practice, there are three CV methods that are traditional approaches, i.e., the sub-sampling test or k-fold cross-validation (k-fold CV), jackknife test, and independent validation test or external test. Among these methods, the jackknife test is recognized as the least arbitrary and most objective one, as mention by Equations (28)–(32) in [75]. Meanwhile, the external test is considered as one of the most rigorous and objective methods for cross-validation in statistics. In the k-fold cross-validation procedure, the training set is randomly separated into k subsets. From the k subsets, a single subset is taken as the testing set to validate the prediction model trained and learned by the remaining k-1 subsets. This process is repeated k times, until each subset had been used as the testing set. During the jackknifing process, a single sample in the whole dataset having N samples is taken as the testing set and the remaining N-1 samples were used for training the model. This process was repeated N times, until each sample was used as the testing set.

In order to evaluate the prediction ability of the model, the following sets of four metrics were used as follows:

(7) $$\mathrm{ACC}=\frac{\mathrm{TP}+\mathrm{TN}}{\left(\mathrm{TP}+\mathrm{TN}+\mathrm{FP}+\mathrm{FN}\right)}$$

(8) $$\mathrm{SN}=\frac{\mathrm{TP}}{\left(\mathrm{TP}+\mathrm{FN}\right)}$$

(9) $$\mathrm{SP}=\frac{\mathrm{TN}}{\left(\mathrm{TN}+\mathrm{FP}\right)}$$

(10) $$\mathrm{MCC}=\frac{\mathrm{TP}\times \mathrm{TN}-\mathrm{FP}\times \mathrm{FN}}{\sqrt{\left(\mathrm{TP}+\mathrm{FP}\right)\left(\mathrm{TP}+\mathrm{FN}\right)\left(\mathrm{TN}+\mathrm{FP}\right)\left(\mathrm{TN}+\mathrm{FN}\right)}}$$

where ACC, SN, SP, and MCC are called the accuracy, sensitivity, specificity, and Matthews coefficient correlation, respectively. TP, TN, FP, and FN represent the instances of true positive, true negative, false positive, and false negative, respectively. Moreover, the receiver operating characteristic (ROC) curves were exploited to evaluate the prediction performance of models using threshold-independent parameters. The area under the ROC curve (auROC) was used to measure the prediction performance, where the area under the curve (AUC) values of 0.5 and 1 were indicative of perfect and random models, respectively.

## 4. Conclusions

A phage virion protein (PVP) plays a crucial role in the research of bacterial infections, especially in bacterial drug resistance. Thus, a computational method that can both effectively predict and analyze PVPs may help researchers in reducing cost as well as the time spent in providing a better understanding of the biochemical and biophysical properties of PVPs. Unfortunately, there is no study so far that provides a systematic effort for the prediction and analysis of PVPs. Herein, we have developed an efficient and interpretable PVP predictor named PVPred-SCM in a systematic manner by taking advantage of a scoring card method (SCM) and the sequence features of propensity scores of amino acids and dipeptides. PVPred-SCM predicts an unknown sequence using the weighted-sum function that can easily be adopted using a single threshold for discriminating PVPs from non-PVPs. Unlike existing methods in pursuit of high prediction performances, PVPred-SCM aims to maximize the interpretability of both classifier and feature usage while maximizing predictive performance of a model. Upon comparing with the state-of-the-art method (PVP-SVM) on the same benchmark dataset, it was found that the performance of PVPred-SCM was comparable to that of PVP-SVM (79.80% vs. 77.66%) as assessed by the rigorous independent validation test, indicating that the proposed model PVPred-SCM is very promising considering its simplicity, interpretability, and implementation. Additionally, the propensity score of amino acids and informative physicochemical properties derived from PVPred-SCM were used to provide insights into the biochemical and biophysical properties of PVPs. Finally, a web server named PVPred-SCM was established and made freely available online at http://camt.pythonanywhere.com/PVPred-SCM. Due to the high potential of our systematic approach employed in this study, PVPred-SCM could be extended for predicting and analyzing many other types of protein and peptide functions without any major modifications, such as predicting HIV-1 CRF01-AE co-receptor usage [18,62], prediction of the human leukocyte antigen gene [30,76], predicting antifreeze proteins [33], predicting the hemolytic activity of peptides [29], predicting anticancer activity of peptides [20], predicting antiviral activity of peptides [40], and predicting antihypertensive activity of peptides [28]. Furthermore, the proposed model could be integrated with other beneficial peptide features such as pseudo amino acid composition [50,77] or amphiphilic pseudo amino acid composition [78] as proposed by Chou [77,79,80,81,82] for further improving the PVP prediction.

## Figures and Tables

**Table 1:** Summary of some existing methods for predicting phage virion proteins.

Method	Classifier ^a^	Sequence Feature ^b^	Independent Test	Webserver
Seguritan et al.’s method [8]	ANN	AAC, PIP	-	-
Feng et al.’s method [9]	NB	AAC, DPC	-	-
PVPred [10]	SVM	g-gap DPC	✓	✓
PVP-SVM [12]	SVM	AAC, DPC, ATC, CTD, PCP	✓	✓
PhagePred [13]	Multinomial NB	g-gap DPC feature tree	-	✓ ^c^
Tan et al.’s method [14]	SVM	GDC	✓	-
Pred-BVP-Unb [16]	SVM	CT, SAAC, bi-PSSM	✓	-
PVPred-SCM (This study)	SCM	DPC	✓	✓

^a^ ANN: artificial neural network, NB: Naïve Bayes, SCM: scoring card method, SVM: support vector machine. ^b^ AAC: amino acid composition, ATC: atomic composition, bi-PSSM: bi-profile position specific scoring matrix, CTD: chain-transition-distribution, CT: composition and translation, DPC: dipeptide composition, g-gap DPC: g-gap dipeptide composition, PCP: physicochemical properties, PIP: protein isoelectric points, SAAC: split amino acid composition. ^c^ The webserver was not functional during our manuscript preparation.

**Table 2:** Comparison of ten SCM models with ten different optimized dipeptide propensity scores (opti-DPS) over 10-fold cross-validation.

#Exp.	Fitness Score	Threshold	ACC (%)	SN (%)	SP (%)	MCC	auROC
1	0.955	443.96	92.50	99.00	89.41	0.849	0.952
2	0.955	459.91	93.15	91.89	93.76	0.851	0.954
3	0.968	471.80	95.11	94.00	95.64	0.894	0.966
4	0.946	476.36	92.15	95.89	90.31	0.840	0.942
5	0.960	455.34	94.44	96.00	93.74	0.882	0.960
6	0.956	458.08	93.16	96.00	91.81	0.856	0.953
7	0.950	446.56	92.50	96.00	90.81	0.846	0.947
8	0.954	446.24	92.51	98.00	89.88	0.849	0.953
9	0.950	461.75	92.52	95.89	90.86	0.846	0.948
10	0.960	463.10	93.82	94.78	93.29	0.866	0.960
Mean	0.955	458.31	93.18	95.74	91.95	0.858	0.954
STD.	0.006	10.77	0.98	1.97	2.05	0.018	0.007

The threshold is an optimal score for discriminating PVPs from non-PVPs. Meanwhile, ACC, SN, SP, MCC, and auROC are accuracy, sensitivity, specificity, Matthews coefficient correlation, and area under the receiver operating characteristic (ROC) curve, respectively.

**Table 3:** Comparison of ten SCM models with ten different optimized dipeptide propensity scores (opti-DPS) over independent validation test.

#Exp.	Fitness Score	Threshold	ACC (%)	SN (%)	SP (%)	MCC	auROC
1	0.955	443.96	74.47	80.00	71.88	0.486	0.782
2	0.955	459.91	75.53	73.33	76.56	0.476	0.743
3	0.968	471.80	76.60	70.00	79.69	0.482	0.781
4	0.946	476.36	76.60	63.33	82.81	0.461	0.775
5	0.960	455.34	76.60	63.33	82.81	0.461	0.793
6	0.956	458.08	71.28	73.33	70.31	0.410	0.749
7	0.950	446.56	72.34	76.67	70.31	0.440	0.749
8	0.954	446.24	70.21	73.33	68.75	0.395	0.742
9	0.950	461.75	77.66	76.67	78.13	0.523	0.781
10	0.960	463.10	73.40	66.67	76.56	0.417	0.787
Mean	0.955	458.31	74.47	71.67	75.78	0.455	0.768
STD.	0.006	10.77	2.56	5.72	5.22	0.040	0.020

The threshold is an optimal score for discriminating PVPs from non-PVPs. Meanwhile, ACC, SN, SP, MCC, and auROC are accuracy, sensitivity, specificity, Matthews coefficient correlation, and area under the ROC curve, respectively.

**Table 4:** Comparison between PVPred-SCM based on optimized (opti-DPS) and initial (init-DPS) dipeptide propensity scores assessed by 10-fold cross-validation and independent validation test.

Method	10-fold CV		Independent Test			
	ACC (%)	MCC	ACC (%)	SN (%)	SP (%)	MCC
Init-DPS	85.99	0.705	75.53	53.33	85.94	0.414
opti-DPS	92.52	0.846	77.66	76.67	78.13	0.523

**Table 5:** Performance comparisons between PVPred-SCM and existing methods as assessed by the 10-fold cross-validation and independent validation test.

Method	10-fold CV		Independent Test			
	ACC (%)	MCC	ACC (%)	SN (%)	SP (%)	MCC
Feng et al.’s method ^a^	79.15	-	-	-	-	-
PVPred ^a^	85.02	-	71.30	60.00	76.50	0.357
PVP-SVM ^a^	87.00	0.695	79.80	66.70	85.90	0.531
PhagePred ^a^	98.05	0.963	-	-	-	-
Tan et al.’s method ^a^	87.95	0.761	75.53	70.00	78.13	0.464
PVPred-SCM	92.52	0.846	77.66	76.67	78.13	0.523

^a^ Results were reported from the work of Tan et al.’s method [14].

**Table 6:** Top ten potential phage virion proteins having the highest of their PVP scores.

Name (Uniprot)	PVP Score	PDBID	UniProtID	Source
Capsid protein G8P	605.69	1HH0	P82889	*Thermus* phage PH75
Capsid protein G8P	581.12	2IFO	P03622	*Xanthomonas* phage Xf
HIS6-pVII fusion protein	541.13		ADR00487	VCSM13 HIS6-pVII modified interference-resistant helper phage
G VIII capsid protein Precursor	538.64	6A7F	NP_040575	Enterobacteria phage Ike
P34	538.52		YP_009639974	Enterobacteria phage PRD1
Major coat protein	534.58	1IFP	NP_040652	*Pseudomonas* phage Pf3
Structural protein P7	532.24		NP_049902	*Pseudoalteromonas* virus PM2
Transclycosylase	529.49		YP_009639979	Enterobacteria phage PRD1
MULTISPECIES: major coat protein	529.45		WP_015979773	Enterobacteriaceae
Hypothetical protein	519.23		WP_015975197	*Salmonella enterica*

**Table 7:** The propensity scores of twenty amino acids to be phage virion proteins (score) along with amino acid compositions (%) of PVPs and non- PVPs.

Amino Acid	PVP (%)	Non-PVP (%)	Difference	Score	*p*-Value
A-Ala	9.98	8.09	1.89(1)	529.50(1)	<0.05
T-Thr	6.90	5.49	1.41(4)	511.43(2)	<0.05
V-Val	8.09	6.39	1.71(3)	506.88(3)	<0.05
G-Gly	8.20	6.42	1.78(2)	506.68(4)	<0.05
S-Ser	7.29	6.10	1.19(5)	504.63(5)	<0.05
Y-Tyr	3.37	3.50	−0.12(13)	479.13(6)	0.571
N-Asn	4.69	4.64	0.05(9)	471.50(7)	0.866
P-Pro	4.13	3.76	0.38(7)	462.15(8)	0.178
Q-Gln	4.18	3.72	0.46(6)	452.83(9)	0.106
I-Ile	6.23	6.06	0.17(8)	443.18(10)	0.629
W-Trp	1.38	1.50	−0.12(12)	442.33(11)	0.408
D-Asp	5.23	5.87	−0.64(16)	435.45(12)	<0.05
C-Cys	0.66	1.05	−0.39(14)	426.58(13)	<0.05
M-Met	2.64	2.73	−0.09(11)	426.15(14)	0.626
F-Phe	3.91	3.91	0.00(10)	423.45(15)	0.989
L-Leu	7.80	8.34	−0.54(15)	395.85(16)	0.160
R-Arg	4.28	5.48	−1.21(18)	383.15(17)	<0.05
H-His	1.04	1.80	−0.76(17)	378.45(18)	<0.05
E-Glu	4.85	7.36	−2.51(19)	358.93(19)	<0.05
K-Lys	5.14	7.81	−2.67(20)	310.90(20)	<0.05

**Table 8:** The three important physicochemical properties (PCPs) derived from PVPred-SCM.

Amino Acid	PS	KOEP990101	Side-Chain [50]	WOLR790101
A-Ala	529.50(1)	−0.04(12)	15(19)	1.12(5)
T-Thr	511.43(2)	0.39(3)	45(15)	−0.02(10)
V-Val	506.88(3)	−0.06(13)	43(16)	1.13(4)
G-Gly	506.68(4)	1.24(1)	1(20)	1.20(1)
S-Ser	504.63(5)	0.15(7)	31(18)	−0.05(11)
Y-Tyr	479.13(6)	0.05(8)	107(2)	−0.23(13)
N-Asn	471.50(7)	0.25(5)	58(11)	−0.83(16)
P-Pro	462.15(8)	0.00(9)	42(17)	0.54(9)
Q-Gln	452.83(9)	−0.02(11)	72(9)	−0.78(14)
I-Ile	443.18(10)	−0.26(17)	57(12)	1.16(3)
W-Trp	442.33(11)	0.21(6)	130(1)	−0.19(12)
D-Asp	435.45(12)	0.27(4)	59(10)	−0.83(17)
C-Cys	426.58(13)	0.57(2)	47(14)	0.59(7)
M-Met	426.15(14)	−0.09(14)	75(6)	0.55(8)
F-Phe	423.45(15)	−0.01(10)	91(4)	0.67(6)
L-Leu	395.85(16)	−0.38(20)	57(13)	1.18(2)
R-Arg	383.15(17)	−0.30(18)	101(3)	−2.55(20)
H-His	378.45(18)	−0.11(15)	82(5)	−0.93(19)
E-Glu	358.93(19)	−0.33(19)	73(7)	−0.92(18)
K-Lys	310.90(20)	−0.18(16)	73(8)	−0.80(15)
Correlation R	1.000	0.502	−0.516	0.484

## Supplementary Materials

The following are available online at https://www.mdpi.com/2073-4409/9/2/353/s1, Table S1. Performance comparisons between PVPred-SCM with existing methods on independent dataset derived from Zhang et al’s method [11]. Table S2. The ten top-ranked informative physicohemical properties having the highest pearson correlation (R) with the propensity scores of amino acids. Table S3. The ten top-ranked informative physicohemical properties having the lowest pearson correlation (R) with the propensity scores of amino acids.
