# Supplementary material for: PVPred-SCM: Improved Prediction and Analysis of Phage Virion Proteins Using a Scoring Card Method
Source: Cells. 2020 Feb 3;9(2):353. doi: 10.3390/cells9020353 (PMC7072630; doi:10.3390/cells9020353)
Supplement: Supplementary file 1 [file cells-09-00353-s001.pdf]

Supplementary files

Table S1. Performance comparisons between PVPred-SCM with existing methods on independent dataset derived from Zhang et al's method [11].

**Method**	**ACC (%)**	**SN (%)**	**SP (%)**	**MCC**
Feng et al's method[a]	67.50	75.00	62.00	0.37
PVPred[a]	72.50	60.30	81.50	0.43
Zhang et al's method [a]	83.10	85.30	81.50	0.66
PVPred-SCM	74.40	72.10	76.10	0.48

[a] Results were reported from the work of Zhang et al's method [11].

Table S2. The ten top-ranked informative physicohemical properties having the highest pearson correlation (R) with the propensity scores of amino acids

Rank	AAindex	Correlation R	Description
**1**	FUKS010107	0.593	Interior composition of amino acids in extracellular proteins of mesophiles (percent) (Fukuchi-Nishikawa, 2001)
**2**	FUKS010111	0.542	Entire chain composition of amino acids in extracellular proteins of mesophiles (percent) (Fukuchi-Nishikawa, 2001)
**3**	JACR890101	0.523	Weights from the IFH scale (Jacobs-White, 1989)
**4**	PRAM820102	0.522	Slope in regression analysis x 1.0E1 (Prabhakaran-Ponnuswamy, 1982)
**5**	QIAN880126	0.521	Weights for beta-sheet at the window position of 6 (Qian-Sejnowski, 1988)
**6**	SNEP660102	0.516	Principal component II (Sneath, 1966)
**7**	KOEP990101	0.502	Alpha-helix propensity derived from designed sequences (Koehl-Levitt, 1999)
**8**	QIAN880124	0.498	Weights for beta-sheet at the window position of 4 (Qian-Sejnowski, 1988)
**9**	RADA880105	0.485	Transfer free energy from vap to oct (Radzicka-Wolfenden, 1988)
**10**	WOLR790101	0.484	Hydrophobicity index (Wolfenden et al., 1979)

Table S3. The ten top-ranked informative physicohemical properties having the lowest pearson correlation (R) with the propensity scores of amino acids

Rank	AAindex	Correlation R	Description
1	HUTJ700102	-0.760	Absolute entropy (Hutchens, 1970)
2	HUTJ700103	-0.720	Entropy of formation (Hutchens, 1970)
3	ZIMJ680103	-0.715	Polarity (Zimmerman et al., 1968)
4	FAUJ880104	-0.695	STERIMOL length of the side chain (Fauchere et al., 1988)
5	LEVM760105	-0.662	Radius of gyration of side chain (Levitt, 1976)
6	FAUJ880111	-0.640	Positive charge (Fauchere et al., 1988)
7	CHAM830104	-0.640	The number of atoms in the side chain labelled 2+1 (Charton-Charton, 1983)
8	LEVM760102	-0.625	Distance between C-alpha and centroid of side chain (Levitt, 1976)
9	GEIM800101	-0.618	Alpha-helix indices (Geisow-Roberts, 1980)
10	EISD860102	-0.584	Atom-based hydrophobic moment (Eisenberg-McLachlan, 1986)
